# Comparative Genomic and Mitochondrial Phylogenetic Relationships of Ovulidae (Mollusca: Gastropoda) Along the Chinese Coast

**DOI:** 10.1002/ece3.71224

**Published:** 2025-04-09

**Authors:** Qiong Wu, Peng Xiang, ShiHao Fan, GuangCheng Chen, BingPeng Xing

**Affiliations:** ^1^ Third Institute of Oceanography Ministry of Natural Resources Xiamen China; ^2^ College of Life Sciences Beijing Normal University Beijing China; ^3^ Independent Researcher Beijing China

**Keywords:** gastropod, mitochondrial genome, molecular phylogeny, Ovulidae

## Abstract

The family Ovulidae, closely related to Cypraeidae (cowries), comprises approximately 260–280 species predominantly inhabiting tropical and subtropical shallow marine environments. Unlike cowries, ovulids have a more specialized diet, with most species feeding on soft corals, leather corals, or black corals. Given their proximity in distribution and close phylogenetic relationship, dietary specialization may have played a crucial role in the evolutionary divergence of cowries and ovulids. However, unlike cowries, the traditional morphological classification of Ovulidae has proven challenging due to substantial variability influenced by their host organisms, leading to ongoing debates regarding species delimitation and taxonomy. In this study, we collected 15 Ovulidae species from China's offshore waters, including the first regional record of *Habuprionovolva aenigma* (M. Azuma & C. N. Cate, 1971). We successfully obtained mitochondrial genomes for 14 of these species and found that, with the exception of *H. aenigma* , they exhibit the typical mitochondrial genome structure of Caenogastropoda. Our phylogenetic analyses based on mitochondrial genome data, along with the discovery of a unique mitochondrial tRNA gene order in the subfamily Prionovolvinae, support recent studies suggesting that the genera *Naviculavolva* and *Contrasimnia* should be reclassified from the Simniinae to the Prionovolvinae. Additionally, our results do not support the monophyly of the genus *Cuspivolva*. Furthermore, our findings indicate that mitochondrial gene rearrangements occurred after the divergence of the subfamilies Prionovolvinae and Ovulinae. Additionally, we found that Ovulidae species display significantly higher Ka/Ks ratios compared to Cypraeidae, indicating different selective pressures, possibly linked to their unique feeding habits. This research enhances the understanding of Ovulidae phylogeny and provides genomic resources for future studies.

## Graphical abstract

This study explores the phylogeny and mitochondrial genomics of Ovulidae, a family of marine gastropods related to Cypraeidae, by analyzing mitochondrial genomes from 14 of 15 species collected off China's coast. Key findings include the reclassification of genera Naviculavolva and Contrasimnia to the subfamily Prionovolvinae, evidence against the monophyly of Cuspivolva, and insights into mitochondrial gene rearrangements and higher Ka/Ks ratios in Ovulidae compared to Cypraeidae, suggesting distinct selective pressures tied to their specialized diets. These results deepen our understanding of Ovulidae evolution and offer valuable mitochondrial genomic resources for future research.

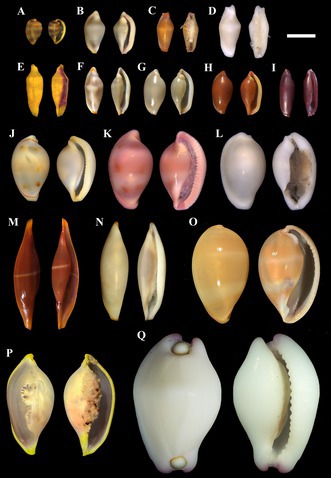

## 1 Introduction

The classification within Cypraeoidea has been in flux, but the family Ovulidae (Superfamily: Cypraeoidea), also known as ovulids, cowry allies, or false cowries, is widely regarded as the closest relative of Cypraeidae (Rosenberg 1992; Schiaparelli et al. 2005). The Ovulidae originated in the early Eocene (Schiaparelli et al. 2005), and currently includes approximately 260–280 extant species (Galli 2024; WoRMS 2011; Zvonareva et al. 2020). Most species within this family inhabit depths of less than 50 m (Lorenz and Fehse 2009), with distributions largely coinciding with the distribution patterns of Cypraeidae, primarily in tropical and subtropical marine regions (Reijnen and van der Meij 2019; Rosenberg 1992).

As close relatives of cowries, ovulids are particularly notable for their specialized feeding habits. Most Ovulidae species feed on octocorals as ectoparasites, while a few consume sponges, crinoids, and antipatharians (Lorenz and Fehse 2009; Reijnen et al. 2010). Furthermore, ovulid species often exhibit a certain degree of host specificity (Reijnen et al. 2010), with some species appearing to prey exclusively on a single host species, while others demonstrate selectivity at the family level (Lorenz and Fehse 2009; Nocella et al. 2024). Evidently, the interactions and co‐evolution between ovulids and their hosts profoundly influence their evolutionary trajectories, from genetic divergence to morphological adaptation (De Baets and Huntley 2021; Lorenz and Fehse 2009). Current evidence suggests that ovulids select their hosts based on stochastic ecological factors. Phylogenetically, there appears to be no clear correlation between ovulids and their hosts at the species level (Schiaparelli et al. 2005); however, at higher taxonomic levels, a strong association between the two is observed (Nocella et al. 2024). Another consequence of their parasitic lifestyle is the variation in their coloration and morphology, which often vary to resemble their hosts, exhibiting significant plasticity that complicates species identification (Lorenz and Fehse 2009; Reijnen and van der Meij 2017; Rosenberg 1992). This high degree of morphological variability has likely contributed to widespread taxonomic confusion within the family. Rosenberg (1992) estimated that the number of valid Ovulidae species might be as low as 160–170, and Nocella et al. (2024) similarly noted that “the inflation of nominal species in the systematics of ovulids can be ascribed to the extreme morphological plasticity of most of the species.” In recent years, several synonymies within Ovulidae have been confirmed. However, due to the lack of molecular data for many species within this family, the number of valid species in Ovulidae remains uncertain.

The family Ovulidae was established by British taxonomist Fleming (1822), who described two genera—*Volva* and *Calpurnus*—in History of British Animals. Early morphological classifications were made by Schilder (1932), Schilder and Schilder (1971), Cate (1973) and Cate (1974). Reflecting Fleming's genera, Ovulidae are generally divided into two subfamilies: Ovulinae and Volvinae (Schiaparelli et al. 2005; Schilder 1932). With the advent of molecular biology, researchers have sought to clarify the phylogenetic relationships within Ovulidae using various genetic markers, including 16S rRNA (Schiaparelli et al. 2005), cox1 (Reijnen et al. 2010), 28S (Reijnen and van der Meij 2019; Zvonareva et al. 2020), histone H3 gene (Reijnen and van der Meij 2019), and ITS1‐5.8S‐ITS2 (Wu et al. 2022). Molecular phylogenetics appears to yield different conclusions from morphological systematics; for example, the study by Schiaparelli et al. (2005) suggests that apart from the subfamily Ovulinae (excluding *Ovula ovum* [Linnaeus, 1758]), the remaining Ovulidae species can be allocated to four subfamilies of uncertain taxonomic status (Reijnen and van der Meij 2019; Schiaparelli et al. 2005). Furthermore, Fehse (2007) expanded on this by dividing Ovulidae into four subfamilies. The most comprehensive and systematic phylogenetic study of Ovulidae to date was conducted by Nocella et al. (2024), which utilized a nuclear gene (28S rDNA) and two mitochondrial genes (cox1 and 16S rRNA) covering 36 genera. Their findings revealed that eight of these genera are not monophyletic. At the subfamily level, Ovulinae and Aclyvolvinae were confirmed to be monophyletic, whereas Simniinae and Prionovolvinae were found to consist of multiple distinct lineages (Nocella et al. 2024).

Mitochondrial genomes have long been employed to infer the phylogenetic relationships of bilaterian animals due to their relatively rapid evolutionary rates (Boore 1999; Wang et al. 2023) and their ability to provide substantial genetic information (Irisarri et al. 2020). Their single‐copy nature reduces the difficulty of assessing homology, while gene rearrangements and duplications can provide additional genetic data for phylogenetic analysis (Boore and Brown 1998). However, as of September 2024, only one mitochondrial genome sequence of an Ovulidae species was available in the GenBank database (*Volva habei*, OR492307).

In this study, we collected 15 Ovulidae species from the offshore waters of China, reporting *Habuprionovolva aenigma* (M. Azuma & C. N. Cate, 1971) for the first time in Chinese waters. We successfully obtained mitochondrial genomes for 14 of these species. By constructing a phylogenetic tree based on mitochondrial genome data for both Ovulidae and Cypraeidae, we examined the phylogenetic relationships among Ovulidae genera distributed along the Chinese coast. Additionally, we conducted a preliminary comparative analysis of selective pressures among species. Through this study, we aim to refine the diversity data of Ovulidae in Chinese coastal waters and, by comparing Ovulidae and Cypraeidae, improve our understanding of host–parasite interactions. Ultimately, our findings provide valuable genomic data to support future research on co‐evolutionary dynamics.

## 2 Materials and Methods

### 2.1 Sample Collection and Identification

Sample collection locations, dates, and methods are detailed in Table 1. Species identification primarily relied on “The Living Ovulidae: A Manual of the Families of Allied Cowries” (Lorenz and Fehse 2009), “Cowries and Their Relatives of China” (Zhang 2011), and “Hardy's Internet Guide to Marine Gastropods” (Hardy 2023). Specimen photographs were captured using a Leica S9D stereo microscope.

**TABLE 1:** Sample collection locations, sampling methods, sampling dates, and GenBank accession numbers.

Subfamily	Species	Sampling locations	Sampling methods	Sampling dates		GenBank accession numbers	Sequence read archive accession(SRA) numbers
				Year	Month		
Prionovolvinae	*Habuprionovolva aenigma*	LingShui, HaiNan, China	Diving (5–10 m)	2022	April	PQ493598	SRR28508812
Prionovolvinae	*Sandalia triticea*	XiaMen, FuJian, China	Intertidal zone	2020	November	PQ459316	SRR30870679
Prionovolvinae	*Crenavolva traillii*	XiaMen, FuJian, China	Intertidal zone	2022	June	PQ493596	SRR28578734
Prionovolvinae	*Cuspivolva bellica*	XiaMen, FuJian, China	Intertidal zone	2020	November	PQ493597	SRR30870678
Prionovolvinae (Simniinae^a^)	*Naviculavolva deflexa*	LingShui, HaiNan, China	Diving (5–10 m)	2021	September	PQ450685	SRR30870681
Prionovolvinae	*Calpurnus verrucosus*	LingShui, HaiNan, China	Diving (5–10 m)	2021	September	PQ450686	SRR30870680
Prionovolvinae	*Cuspivolva queenslandica*	ShiShi, FuJian, China	Diving (5–10 m)	2020	September	PQ516699	SRR30892248
Prionovolvinae (Simniinae^a^)	*Contrasimnia xanthochila*	East China Sea	Trawl (150–200 m)	2023	April	PQ436352	SRR30892246
Prionovolvinae	*Prionovolva brevis*	East China Sea	Trawl (150–200 m)	2023	April	PQ493599	SRR30892245
Prionovolvinae	*Primovula formosa*	Xiamen, Fujian, China	Intertidal zone	2022	June	PQ459317	SRR30892243
Prionovolvinae	*Procalpurnus lacteus*	LingShui, HaiNan, China	Diving (5–10 m)	2022	April	PQ326985 (cox1) PQ326987 (16S rRNA)	SRR30892242
Prionovolvinae	*Prosimnia semperi*	LingShui, HaiNan, China	Diving (5–10 m)	2022	April	PQ493600	SRR30892241
Prionovolvinae	Diminovula alabaster	NingDe, FuJian, China	Collection from aquaculture rafts	2024	August	PQ436351	SRR30892240
Ovulinae	Ovula ovum	LingShui, HaiNan, China	Diving (5–10 m)	2024	March	PQ459314	SRR30892244
Ovulinae	Phenacovolva rosea	XiaMen, FuJian, China	Intertidal zone	2024	May	PQ459315	SRR30892247

^a^ Taxonomy based on the classification system of Felix Lorenz and Fehse (2009).

### 2.2 DNA Extraction and Sanger Sequencing

DNA extraction was performed following the animal tissue extraction protocol of the DNeasy Blood & Tissue Kit (QIAGEN). After extraction, the nucleic acid concentration of the DNA extracts was measured using a BioDrop spectrophotometer. Due to the difficulty of separating polysaccharide components from tissues using the kit, the DNA was diluted to 0.2 μg/mL to mitigate the inhibitory effects of polysaccharides on PCR reactions (Wu et al. 2022). Each 25 μL PCR reaction consisted of 12.5 μL PCR mixture (Taq Plus Master Mix II (Dye Plus)), 1 μL of each primer (10 μM), 2.5 μL diluted DNA extract, and 8 μL ultra‐pure water. The cox1 amplification primers were dgLCO: 5′‐GGT CAA CAA ATC ATA AAG AYA TYG G and dgHCO2198: 5′‐TAA ACT TCA GGG TGA CCA AAR AAY CA (Meyer 2003). The annealing temperature was set at 45°C, increasing by 0.5°C per cycle for 15 cycles, followed by 49°C for 20 cycles. PCR products ranging from 650 to 750 bp in length were collected. The 16S rRNA amplification primers were 16Sar: 5′‐CGC CTG TTT ATC AAA AAC AT and 16Sbr: 5′‐CCG GTC TGA ACT CAG ATC ACG T (Hillis et al. 1996), with an annealing temperature of 52°C, targeting a product length of 500–600 bp. All qualified PCR products were sent to Sangon Biotech (Shanghai) Co. Ltd. for sequencing. The obtained sequences were aligned, and low‐signal‐strength ends were trimmed using SeqMan v. 7.1.0 (DNAStar, USA). The final trimmed cox1 sequences were approximately 680 bp in length, while 16S sequences were approximately 550 bp.

### 2.3 Library Preparation and Next‐Generation Sequencing (NGS)

Total genomic DNA was sent to Majorbio Bio‐pharm Technology Co. Ltd. (Shanghai, China) and Novogene Co. Ltd. for library preparation and high‐throughput sequencing. Libraries were prepared with an average fragment size of approximately 300 bp. The DNA libraries were sequenced on the Illumina Novaseq platform using paired‐end reads of 150 bp. The quality of the raw sequencing data was assessed using Fastp (Chen et al. 2018).

### 2.4 Mitochondrial Genome Assembly and Annotation

Mitochondrial genome assembly was performed using NOVOPlasty 4.3.5 (Dierckxsens et al. 2017) with the cox1 or 16S rRNA sequences obtained from Sanger sequencing as seeds, as well as MitoZ (Meng et al. 2019). If circularization was unsuccessful, SeqMan Pro (DNAStar) (Burland 1999) was used to concatenate results from different software. Annotation was conducted using MitoZ (Meng et al. 2019) and the MITOS2 web server (Bernt et al. (2013); Galaxy server: usegalaxy.eu). Protein‐coding gene (PCG) boundaries were confirmed by comparison with closely related species, and the positions of start and stop codons were verified using SnapGene (GLS Biotech). A mitochondrial circular map was generated using Proksee (Grant et al. 2023, https://proksee.ca/). The linearized mitochondrial gene arrangement patterns were generated using PhyloSuite (Zhang et al. 2020) and visualized with iTOL (Letunic and Bork 2021, https://itol.embl.de/).

### 2.5 Phylogenetic Analysis

Given the close relationship between Cypraeidae and Ovulidae, we included 11 Cypraeidae species in the analysis, whose distribution areas largely overlap with those of the ovulids collected in this study (Ma et al. 2023). Two species from the family Tonnidae were selected as outgroups (Li et al. 2024; Pu et al. 2019). Using PhyloSuite (Zhang et al. 2020), 13PCGs and two rRNA genes were extracted. Multiple sequences were aligned with MAFFT (Katoh and Standley 2013) using the “auto” strategy and codon alignment for PCGs or normal alignment for rRNA. The PCG sequences were refined using MACSE (Ranwez et al. 2018), and ambiguously aligned fragments were removed with Gblocks (Talavera and Castresana 2007). Different gene sequences were concatenated using PhyloSuite (Zhang et al. 2020). Two datasets (PCGs, PCGs + rRNA) were used to construct phylogenetic trees. The best‐fit partition model was selected for IQ‐TREE (Nguyen et al. 2015) using the AIC criterion with ModelFinder (Kalyaanamoorthy et al. 2017) and for MrBayes (Ronquist et al. 2012) using the BIC criterion, with the codon model chosen for PCGs.

Bayesian inference (BI) phylogenies were constructed using MrBayes 3.2.6 (Ronquist et al. 2012) under a partition model (two parallel runs, 2,000,000 generations), with the initial 25% of sampled data discarded as burn‐in. Maximum likelihood (ML) phylogenies were inferred using IQ‐TREE (Nguyen et al. 2015) with an edge‐linked partition model for 2000 ultrafast bootstraps (Minh et al. 2013) and the Shimodaira–Hasegawa‐like approximate likelihood‐ratio test (Guindon et al. 2010).

Only species with complete sequences for all 13 PCGs were selected for selection pressure analysis, along with Cypraeidae cypraeid sequences obtained from GenBank. Ka/Ks ratios (nonsynonymous to synonymous substitution rates) were calculated using MEGA with the Jukes‐Cantor model (Nei and Gojobori 1986; Tamura et al. 2021). For Ovulidae species, the Ka/Ks ratio for each PCG was calculated in relation to other species within the family, while for Cypraeidae species, no individual statistics were shown. The K2P distance matrix for the cox1 gene was produced by MEGA.

## 3 Results

**FIGURE 1:**
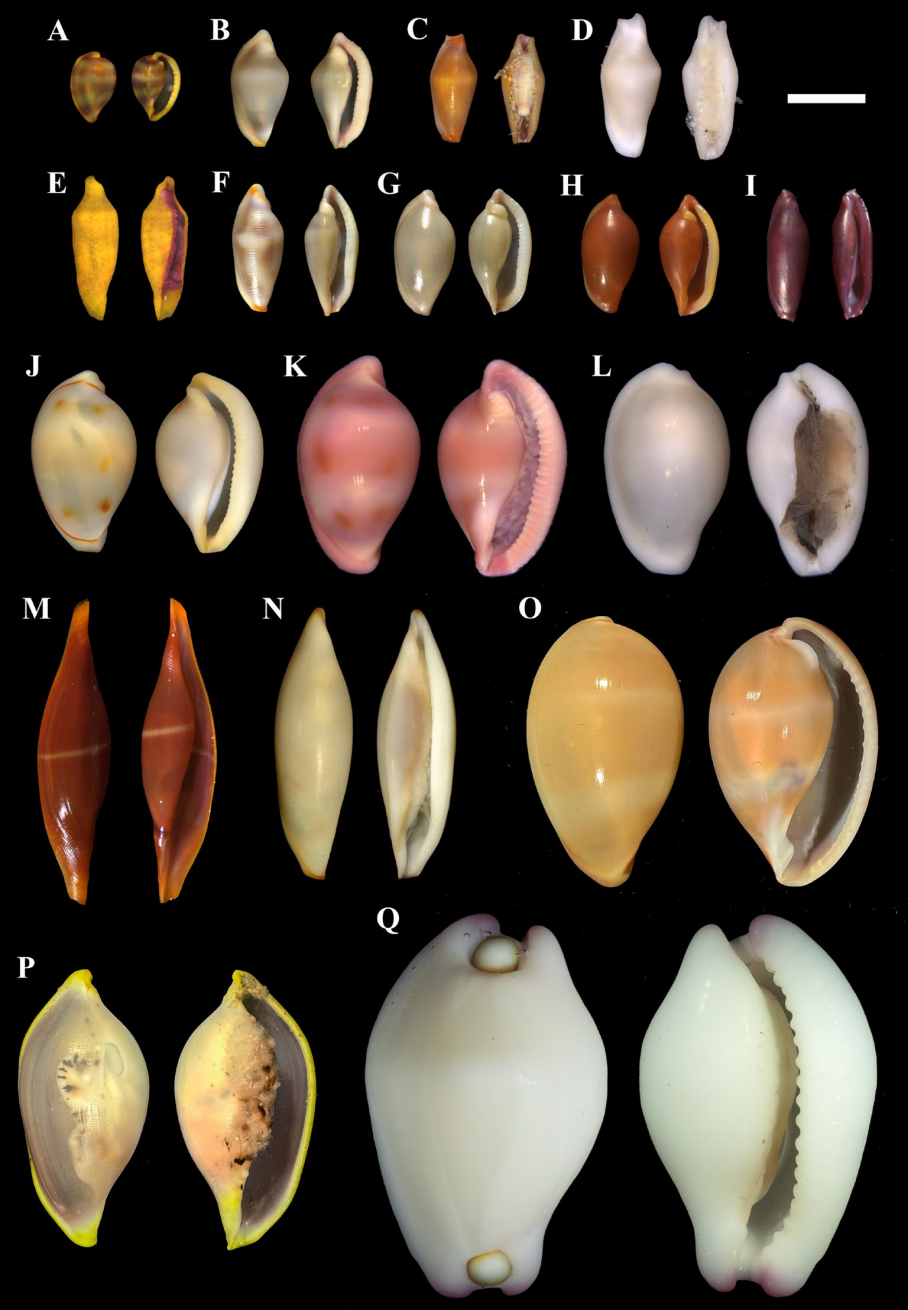
Samples of ovulids: (A) *Habuprionovolva aenigma*; (B) *Cuspivolva bellica*; (C, D) *Crenavolva traillii*; (E) *Prosimnia semperi*; (F) *Primovula formosa*; (G, H) *Sandalia triticea*; (I) *Cuspivolva queenslandica*; (J, K) *Diminovula alabaster*; (L) *Procalpurnus lacteus*; (M) *Phenacovolva rosea*; (N) *Naviculavolva deflexa*; (O) *Prionovolva brevis*; (P) *Contrasimnia xanthochila*; (Q) *Calpurnus verrucosus*.

**FIGURE 2:**
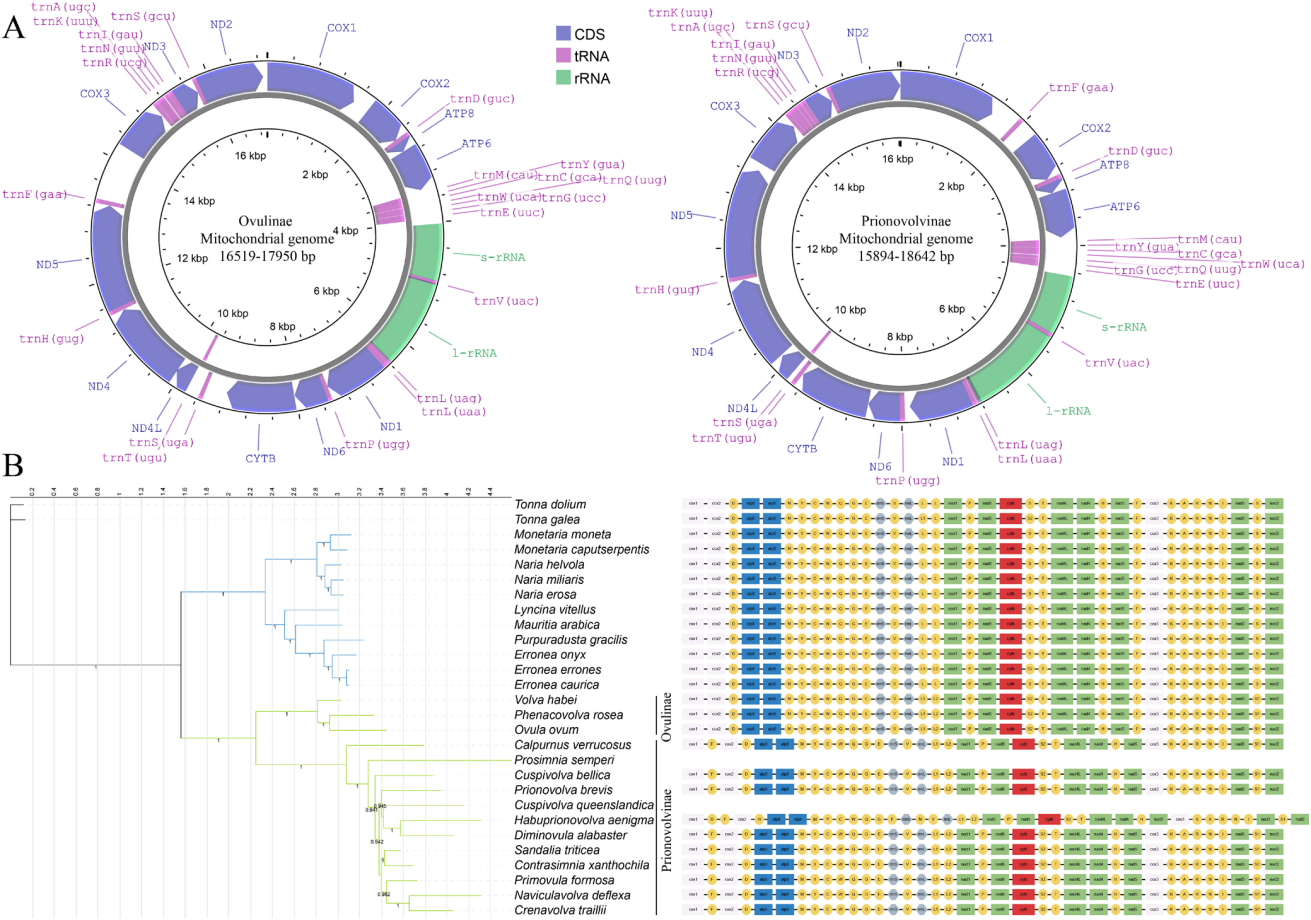
(A) The complete mitochondrial genomes of the subfamilies Ovulinae (PQ459314, PQ459315 and OR492307) and Prionovolvinae (including the genera *Naviculavolva* and *Contrasimnia*, PQ436351–PQ436352, PQ450685–PQ450686, PQ459316–PQ459317, PQ493596–PQ493600, PQ516699) (B) BI mitophylogeny (based on concatenated dataset of 12 PCGs and 2 rRNA genes) and gene order for families Ovulidae and Cypraeidae. Numbers near the nodes are posterior probability (only those > 75% are shown).

Among all the collected Ovulidae specimens (Figure 1), *Procalpurnus lacteus* (Lamarck, 1810) did not yield contigs longer than 1000 bp and was therefore omitted from subsequent analyses. *Cuspivolva queenslandica* (Cate 1974) and *Prosimnia semperi* (Weinkauff, 1881) only produced incomplete mitochondrial genome fragments. Additionally, approximately 500 bp of the l‐rRNA sequence was missing for *Prionovolva brevis* (G. B. Sowerby I, 1828). As a result, 14 Ovulidae species from 13 genera were included in the analysis, along with the mitochondrial genome sequence of *Volva habei* Oyama, 1961, retrieved from GenBank. The mitochondrial genome lengths ranged from 15,894 to 18,642 bp. With the exception of *Habuprionovolva aenigma*, the mitochondrial genomes of the other Ovulidae species comprised two rRNAs, 13 protein‐coding genes, and 22 tRNAs (Figure 2A).

The details of the best partition models are provided in Table S1. The phylogenetic trees for the Cypraeidae portion, constructed using two datasets and two methods, were completely consistent. In the Ovulidae portion, apart from the ML tree for the 13 PCGs, the remaining three phylogenetic trees showed similar topologies (Figure 2B; Figure S1), albeit with minor differences: the BI and ML phylogenetic trees for the 13 PCGs + 2 rRNAs were nearly identical, differing only in the positioning of the crown group comprising *Sandalia triticea* (Lamarck, 1810) and *Contrasimnia xanthochila* (Kuroda, 1928). The ML tree for the 13 PCGs + 2 rRNAs and the BI tree for the 13 PCGs differed only in the interchange of *Cuspivolva queenslandica* and *Prionovolva brevis*. All three phylogenetic trees strongly supported the sister group relationship between *Sandalia triticea* and *Contrasimnia xanthochila*, while the topology of the ML tree for the 13 PCGs differed but showed low support at the nodes.

The mitochondrial gene arrangement results (Figure 2B) revealed that all analyzed Cypraeidae species shared the same gene count and arrangement. Among members of the subfamily Ovulinae, *Ovula ovum*, *Volva habei*, and *Phenacovolva rosea* exhibited the same gene order as Cypraeidae. In contrast, the remaining species (traditionally classified under the subfamilies Prionovolvinae and Simniinae) had trnF located between cox1 and cox2. *Habuprionovolva aenigma* exhibited unique characteristics, with a trnD between cox1 and trnF, and a trnM between rrnS and trnV (Figure 2B). Overall, the mitochondrial genomes of Ovulidae and Cypraeidae displayed similar PCG arrangements and distribution patterns: with the exception of eight tRNAs, the remaining genes were primarily located on the major strand.

**FIGURE 3:**
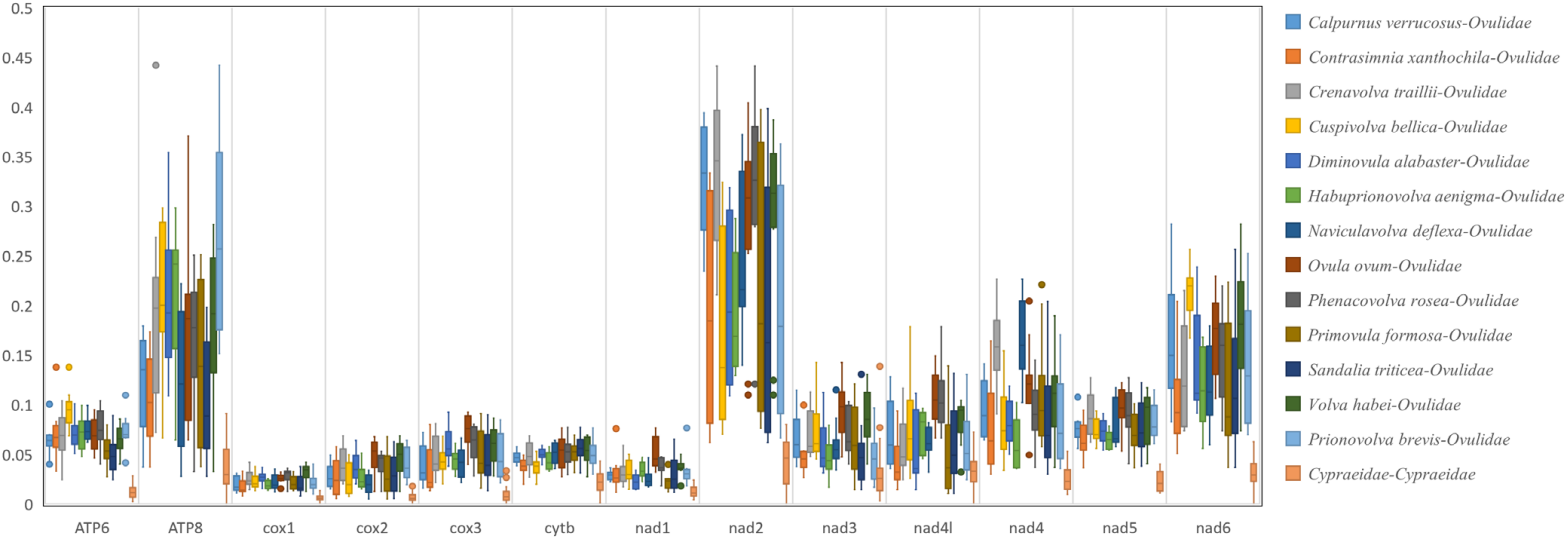
Box plot for pairwise divergence of Ka/Ks ratio (mean ± SD, and range) for 12 PCGs of ovulids and cypraeids mitogenomes.

The Ka/Ks analysis indicated that, while all analyzed species had Ka/Ks values < 1, there were significant differences between the Ka/Ks values of cowries and ovulids, as detailed in Figure 3. Among the analyzed ovulids, the minimum K2P distance was 10.94% (between *Sandalia triticea* and *Prionovolva brevis*), and the maximum was 23.75% (between *Ovula ovum* and *Diminovula alabaster* [Reeve, 1865]), with an average K2P genetic distance of 17.11% across the 14 Ovulidae species, as summarized in Table 2.

**TABLE 2:** K2P interspecific genetic distance matrix of cox1 sequences among 14 species of Ovulidae.

No.	Species	*Calpurnus verrucosus*	*Contrasimnia xanthochila*	*Crenavolva traillii*	*Cuspivolva bellica*	*Cuspivolva queenslandica*	*Diminovula alabaster*	*Habuprionovolva aenigma*	*Naviculavolva deflexa*	*Ovula ovum*	*Phenacovolva rosea*	*Primovula formosa*	*Prionovolva brevis*	*Sandalia triticea*	*Volva habei*
1	*Calpurnus verrucosus*														
2	*Contrasimnia xanthochila*	0.15987194													
3	*Crenavolva traillii*	0.149029437	0.136957596												
4	*Cuspivolva bellica*	0.152876567	0.149998263	0.143921104											
5	*Cuspivolva queenslandica*	0.146811088	0.139665796	0.136216185	0.140778335										
6	*Diminovula alabaster*	0.178148319	0.174831929	0.171523666	0.170588622	0.181696181									
7	*Habuprionovolva aenigma*	0.189917814	0.184068507	0.161631842	0.164384327	0.162728198	0.192677902								
8	*Naviculavolva deflexa*	0.17240009	0.156698631	0.137648682	0.169442505	0.154581678	0.200477566	0.191863271							
9	*Ovula ovum*	0.208910582	0.222816859	0.207562201	0.221882279	0.220006364	0.237548707	0.235204824	0.210153203						
10	*Phenacovolva rosea*	0.207040432	0.212158386	0.201876937	0.218316774	0.212703428	0.221704703	0.230717985	0.203042363	0.153420005					
11	*Primovula formosa*	0.150644889	0.118661708	0.126507142	0.139941515	0.127044253	0.172641572	0.148344417	0.148482582	0.212813862	0.206063174				
12	*Prionovolva brevis*	0.141886166	0.122741074	0.12807581	0.132537306	0.13198369	0.162728198	0.164709541	0.149543686	0.219712927	0.207436312	0.12511341			
13	*Sandalia triticea*	0.132575475	0.117381214	0.111275702	0.134365262	0.115096699	0.163217471	0.157397443	0.135435082	0.214182917	0.198051308	0.109425545	0.101135628		
14	*Volva habei*	0.199377193	0.199711046	0.180677238	0.191043076	0.198118796	0.212235731	0.228047573	0.197651475	0.147434865	0.135435082	0.193912449	0.182485063	0.179530672	

## 4 Discussion

### 4.1 Species Diversity of Ovulidae in China

Compared to Cypraeidae, Ovulidae are a relatively young group, with the oldest fossils dating back only to 56.0–47.8 Ma (Dolin and Ledon 2002). In contrast to the fossil record of Cypraeidae, which is more extensive, the fossils of Ovulidae are fewer and mainly concentrated in the genus *Simnia*. This may be related to the later emergence of Ovulidae and the more fragile nature of their shells, which makes fossil preservation more challenging. Genera with fossil records are rarely distributed in China; therefore, we were unable to locate suitable specimens for dating.

According to Zhang (2011), a total of 71 Ovulidae species have been recorded along the Chinese coast. This number was later expanded with the first record of *Cuspivolva bellica* (Cate 1973) in 2019 (Chen et al. 2022) and the first collection of the genus *Naviculavolva* in China in 2022 (Wu et al. 2023). In this study, we further document the first occurrence of *Habuprionovolva aenigma* in Lingshui, Hainan, China (Figure 1A), bringing the total number of known Ovulidae species in China to 74.

Unfortunately, we were unable to collect certain lineages closely related to cowries, such as the genera *Simnia* and *Aclyvolva*. Consequently, the Ovulidae species included in our phylogenetic analysis belong to three major lineages that are more distantly related to cowries: Ovulinae, Prionovolvinae, and Simniinae (Table 1). Notably, the genus *Naviculavolva* was traditionally classified within Simniinae based on morphological characteristics (Fehse 2007). However, molecular evidence suggests that it is more closely related to the subfamily Prionovolvinae (Nocella et al. 2024).

### 4.2 Phylogenetic Tree and Taxonomic Controversies

The instability in tree topology makes determining which phylogenetic tree is more reliable a persistent challenge. This issue has remained unresolved in previous phylogenetic reconstructions based on different molecular markers and evolutionary models (Nocella et al. 2024; Schiaparelli et al. 2005). Although the inclusion of a greater number of genes in this study has led to higher support values for the phylogenetic tree nodes, inconsistencies in tree structure were still observed across the four phylogenetic trees we constructed. This instability may stem from insufficient divergence among their most common recent ancestors (MRCA), suggesting minimal genetic differences, likely resulting from rapid diversification that may have occurred approximately 13 Myr ago, according to Nocella et al. (2024). Additionally, some studies suggest that inadequate taxon sampling may also contribute to this phenomenon (Mynard et al. 2023; Pollock et al. 2002).

The differences between the ML and BI phylogenetic trees constructed using 13 PCGs primarily concern the topological relationships among *Cuspivolva queenslandica*, *Contrasimnia xanthochila*, *Sandalia triticea*, and *Cuspivolva bellica*. However, both trees did not support the classification of *Cuspivolva queenslandica* and *Cuspivolva bellica* within the same genus. Since our analysis included only two species from the genus *Cuspivolva*, we cannot definitively determine which species require taxonomic revision. Notably, Nocella et al. (2024) also found that *Cuspivolva queenslandica* and *C. cuspis* do not belong to the same genus. Instead, *C. queenslandica* appears to be more closely related to *Sandalia triticea*, while *C. cuspis* is more closely related to *Prionovolva brevis*. Furthermore, some shell collectors, such as Kijineko (2024), have proposed that *C. queenslandica* may be more closely related to the genus *Primovula* (飯野 et al. 2010). Based on these findings, we lean toward reassigning *C. queenslandica* to a genus outside *Cuspivolva*, and a comprehensive systematic review of the genus's validity may be warranted.

Given the structural discrepancies in the phylogenetic trees generated in this study from different datasets and methods, the following discussion focuses on clades that are consistent and exhibit high confidence across the various phylogenetic reconstructions. The phylogenetic tree reconstructed in this study supports several key findings reported in previous research. First, our results confirm the phylogenetic positions of *Ovula ovum*, *Volva habei*, *Phenacovolva rosea*, and *Calpurnus verrucosus*. Fehse (2007) classified these genera, along with *Calcarovula*, *Kurodavolva*, *Pellasimnia*, *Takasagovolva*, and *Xandarovula*, into the subfamily Ovulinae. Second, the close relationship between *Habuprionovolva aenigma* and *Diminovula alabaster* is also corroborated, consistent with findings from Fehse (2007), Schiaparelli et al. (2005) and Nocella et al. (2024). This relationship is further supported by their shared host preference, as both species feed on *Scleronephthya* sp. Third, our analysis reveals a close relationship between *Naviculavolva* and *Crenavolva*, which aligns with the findings of Nocella et al. (2024). This challenges the monophyly of the subfamily Simniinae as defined by Fehse (2007). Additionally, the phylogenetic position of *Contrasimnia xanthochila* also refutes the monophyly of the subfamily Simniinae. Since this study does not include lineages more closely related to cowries, such as the genus *Simnia*, we cannot fully assess the broader applicability of the current definition of the subfamily “Simniinae” However, based on the evidence gathered in this study and the findings of Nocella et al. (2024), both *Naviculavolva* and *Contrasimnia* should be reclassified into the subfamily Prionovolvinae, which forms an independent clade with a distinct tRNA arrangement.

This study also presents several findings that differ from previous research. The phylogenetic tree reconstructed by Nocella et al. (2024) suggests that the genera *Volva* and *Ovula* are sister groups, with *Phenacovolva* as the outgroup. In contrast, our results indicate that *Phenacovolva rosea* and *Ovula ovum* form a sister‐group relationship, a pattern that is consistently supported across all four phylogenetic trees constructed in this study. Furthermore, our analysis reveals that the genus *Primovula* (represented by *Primovula formosa*) clusters with *Naviculavolva* and *Crenavolva*, excluding *Prosimnia semperi*. This result contradicts previous findings by Reijnen and van der Meij (2019) and Nocella et al. (2024). All phylogenetic trees in our study strongly support a sister‐group relationship between *Crenavolva traillii* and *Naviculavolva deflexa*, although phylogenetic trees based on PCGs exhibit lower support for the precise placement of *Primovula formosa*. Additionally, our results indicate that the branch containing *Prosimnia semperi* diverged earlier than those of *Sandalia*, *Diminovula*, and *Habuprionovolva*, a conclusion that is consistently supported across all four phylogenetic trees reconstructed in this study.

### 4.3 Mitochondrial Genomic Characteristics and Gene Order

The PCG sequences of the mitochondrial genomes in Cypraeidae and Ovulidae exemplify the typical mitochondrial gene order found in Caenogastropoda (Li et al. 2024; Wang et al. 2017). The uniform tRNA arrangement across various Cypraeidae species suggests a lack of gene rearrangement events, possibly due to the conservative nature of their mitochondrial sequences or the limited range of genera studied. *Tonna galea* and *Tonna dolium*, serving as outgroups, exhibit the same gene order as Cypraeidae, suggesting that this gene arrangement may represent a synapomorphic trait of this clade. Three Ovulidae species—*Ovula ovum*, *Volva habei*, and *Phenacovolva rosea*—share the same gene order as that observed in Cypraeidae (Figure 2). Based on their phylogenetic placements, we infer that mitochondrial gene rearrangements in Ovulidae likely occurred after the divergence of the subfamilies Prionovolvinae and Ovulinae, as defined by Nocella et al. (2024), which is estimated to have taken place between 42.7 and 30.25 million years ago.

Mitochondrial gene evolution is closely linked to metabolic demands, and known factors influencing mitochondrial genome evolution include low‐energy diets, large body size, cold temperatures, and hypoxic conditions (Yang et al. 2019). Since species from Prionovolvinae and Ovulinae do not exhibit significant differences in distribution range or depth, the mitochondrial gene rearrangements in Prionovolvinae may be associated with dietary preferences. The specialized feeding habits of Ovulinae may reflect an element of active selection: discussions with aquarists during sample collection suggested that these three Ovulinae species could adapt their diets through domestication, whereas other species demonstrated a strong dietary specialization toward octocorals.

### 4.4 Selective Pressure Analysis

The Ka/Ks analysis revealed that the 13 PCGs of the studied 13 genera exhibited significantly higher Ka/Ks values than those of Cypraeidae (Figure 3). Factors potentially influencing selection pressure on mitochondrial PCGs include diet, climate (Mishmar et al. 2003), generation time, locomotion capabilities, and effective population size (Jakovlić et al. 2023). Given that *Ovula ovum* and *Volva habei* are of medium size and *Calpurnus verrucosus* is similar in size to smaller Cypraeidae, the differences in Ka/Ks values cannot be solely attributed to size. Additionally, since the Cypraeidae and Ovulidae species are geographically proximate, temperature differences are also unlikely to explain the variations. We suggest that the unique feeding habits of Ovulidae may contribute to some of the Ka/Ks discrepancies, potentially reducing the purifying selection pressure on their mitochondria (Jakovlić et al. 2023).

Notably, there appears to be a pattern in the distribution of the Ka/Ks ratios for mitochondrial genes, particularly for the nad2 gene, which may correlate with species size or population scale (Jakovlić et al. 2023). The species with the five highest median Ka/Ks values for nad2 are *Crenavolva traillii*, *Calpurnus verrucosus*, *Phenacovolva rosea*, *Volva habei*, and *Ovula ovum*, all significantly higher than the others. Except for *Crenavolva traillii*, these four are also the largest among the samples and have close phylogenetic relationships. Regarding population size, as noted by Bouchet (2009), quantitative data for most mollusks are lacking. However, driven by shell specimen trade, common Ovulidae species tend to have stable market prices, which, when controlling for sampling and logistics costs, could serve as a proxy for collection difficulty and population size. According to Kijineko's Cowries Collection (www.cypraea.jp), the two most expensive species are *Cuspivolva bellica* and *Primovula formosa*, with distribution areas and depths comparable to those of *Crenavolva traillii* and *Sandalia triticea* (white individuals), suggesting potentially smaller populations. Correspondingly, these two species have lower median nad2 Ka/Ks values. Given the multifactorial influences on Ka/Ks ratios, and the close phylogenetic relationships among several large ovulids, a larger‐scale study encompassing various environmental factors is necessary to ascertain the determinants of Ka/Ks ratios.

### 4.5 Genetic Distance and Ecological Traits

The genetic distance between closely related species is influenced by various factors, including divergence time (Wayne et al. 1991), generation length (Thomson et al. 2014), ecological factors (Lee and Mitchellles 2011; Zhao et al. 2018), climatic and geographical conditions (Lee and Mitchellles 2011; Zhao et al. 2018), and effective population size (Woolfit 2009). Species with closer genetic distances often exhibit more similar ecological characteristics (Burns and Strauss 2011) and morphological traits (Losos 2011).

In this study, the genetic distances among ovulid species do not appear to be driven by a single factor. Within the subfamily Ovulinae, phylogenetic relationships inferred from morphological differences align considerably with the molecular phylogenetic tree and genetic distance matrix. The K2P genetic distance results indicate that *Phenacovolva rosea* and *Volva habei* are more closely related among the three Ovulinae species (Table 2), both characterized by exhibiting elongated anterior and posterior terminals. Following them, *Ovula ovum* and *Volva habei* are distinguished by their large sizes, which rank among the largest within the family Ovulidae. From the phylogenetic tree, possessing elongated terminals appears to be a plesiomorphic trait for this clade, as other genera within this lineage, such as *Pellasimnia* and *Takasagovolva*, also exhibit similar characteristics. However, *Ovula* seems to be an exception to this trend. In contrast, the situation within the subfamily Prionovolvinae appears more complex. Notably, we were surprised to find that *Sandalia triticea* and *Prionovolva brevis* exhibit a close genetic distance. Apart from a potential partial overlap in distribution range, there are no apparent similarities in their depth distribution, body size, morphology, or host preference that would indicate a close relationship. In contrast, *Primovula formosa*, which was collected from a region highly overlapping with *Sandalia triticea* and sharing the same host species, exhibits a genetic distance consistent with this ecological and size similarity. Given the placement of *Sandalia triticea* in the phylogenetic tree—where it forms a crown group with *Contrasimnia xanthochila*, a species commonly found at depths around 200 m—and shows a close relationship with *Prionovolva brevis*—it is plausible that the ancestors of *Sandalia triticea* originated in relatively deeper waters (at depths of 100 m or more) before expanding into intertidal zones. Both *Prionovolva brevis* and *Contrasimnia xanthochila* possess relatively larger body sizes compared to *Sandalia triticea*, and thin, inflated, and rounded dorsal shells, suggesting that the deep‐water ancestors of *Sandalia triticea* may have shared similar characteristics. Through the process of evolutionary adaptation, *Sandalia triticea* appears to have acquired strong adaptive capabilities, enabling it to thrive on different hosts (*Hicksonella* sp. and *Melitodes flabellifera* [Kükenthal, 1908] [Ma 1997]) and across a broad depth range from the intertidal zone to 200 m (F. Lorenz, 2009; *S. bridgesi* is a synonym of *S. triticea* [Wu et al. 2022]). Additionally, it is the most cold‐tolerant ovulid species recorded in China, with its northernmost intertidal distribution reaching near Qingdao (approximately 36° N). Furthermore, this species appears to exhibit some degree of tolerance to low‐salinity environments, as we have also collected specimens near estuarine regions.

We acknowledge the limitations of relying solely on mitochondrial genomes to reconstruct phylogenetic trees. Factors such as incomplete lineage sorting (Kimball et al. 2021; McGuire et al. 2007), introgression, maternal inheritance, and recombination can complicate mtDNA interpretations (Rubinoff et al. 2006), making it less suitable as the sole data source for phylogenetic analysis. On the other hand, compared to nuclear genomes, mitochondrial genomes evolve more rapidly due to their haploid nature (DeSalle et al. 2017) and exhibit greater variability at lower taxonomic levels (Kelava et al. 2024). Mitochondrial phylogenetic trees can reflect true species relationships more accurately than individual nuclear markers (Kimball et al. 2021).

In this study, despite utilizing complete mitochondrial genomes, some nodes in the phylogenetic trees remain unresolved. The rarity of fossil evidence, the instability of morphological traits, and the variability in host–parasite relationships (Schiaparelli et al. 2005) suggest that molecular techniques may be the best approach to resolving the phylogenetic relationships within this group. Future research should focus on acquiring additional genetic markers, such as nuclear genes, and exploring approaches to integrating nuclear and mitochondrial data to construct more reliable phylogenetic trees (Fisher‐Reid and Wiens 2011).

## 5 Conclusion

In this study, we obtained complete mitochondrial genomes for 12 species of the Ovulidae and partial mitochondrial genome fragments for two additional species. Using two datasets, we reconstructed phylogenetic trees using Bayesian Inference and Maximum Likelihood methods. We suggest reclassifying the genera *Naviculavolva* and *Contrasimnia* from the subfamily Simniinae to the subfamily Prionovolvinae. Additionally, *Cuspivolva queenslandica* should be removed from the genus *Cuspivolva*, and a comprehensive taxonomic revision of *Cuspivolva* is warranted. Subfamily Ovulinae, which is closely related to Cypraeidae, shares the same mitochondrial gene order as Cypraeidae species, as seen in *Volva habei*, *Phenacovolva rosea*, and *Ovula ovum*. In contrast, species in the reclassified subfamily Prionovolvinae, except for *Habuprionovolva aenigma*, exhibit a consistent and characteristic mitochondrial gene order for this subfamily. Furthermore, we compared the selective pressures on mitochondrial protein‐coding genes between different species of Ovulidae and Cypraeidae, finding that Cypraeidae species experience significantly higher selective pressures. Finally, we analyzed genetic distances among various Ovulidae species.

## Author Contributions

**Qiong Wu:** conceptualization (lead), data curation (equal), investigation (supporting), methodology (equal), software (equal), validation (equal), visualization (equal), writing – original draft (lead). **Peng Xiang:** funding acquisition (equal), resources (equal), supervision (equal). **ShiHao Fan:** investigation (equal), writing – review and editing (equal). **GuangCheng Chen:** funding acquisition (equal), resources (equal). **BingPeng Xing:** funding acquisition (lead), investigation (equal), project administration (lead), resources (equal), supervision (equal), writing – review and editing (equal).

## Conflicts of Interest

The authors declare no conflicts of interest.

## Supporting information


**Figure S1.** Mitophylogeny for families Ovulidae and Cypraeidae. Numbers near the nodes are posterior probability/bootstraps values (only those > 75% are shown). (A) ML phylogenetic tree (13 PCGs + 2 rRNAs) (B) ML phylogenetic tree (13 PCGs) (C) BI phylogenetic tree (13 PCGs).

**Table S1.** The best‐fit model of nucleotide substitution.

## Data Availability

All sequencing data generated in this study are available in Table 1.
