# Supplementary figures and images for: Comparative Genomic and Mitochondrial Phylogenetic Relationships of Ovulidae (Mollusca: Gastropoda) Along the Chinese Coast
Source: Ecol Evol. 2025 Apr 9;15(4):e71224. doi: 10.1002/ece3.71224 (PMC11981875; doi:10.1002/ece3.71224)

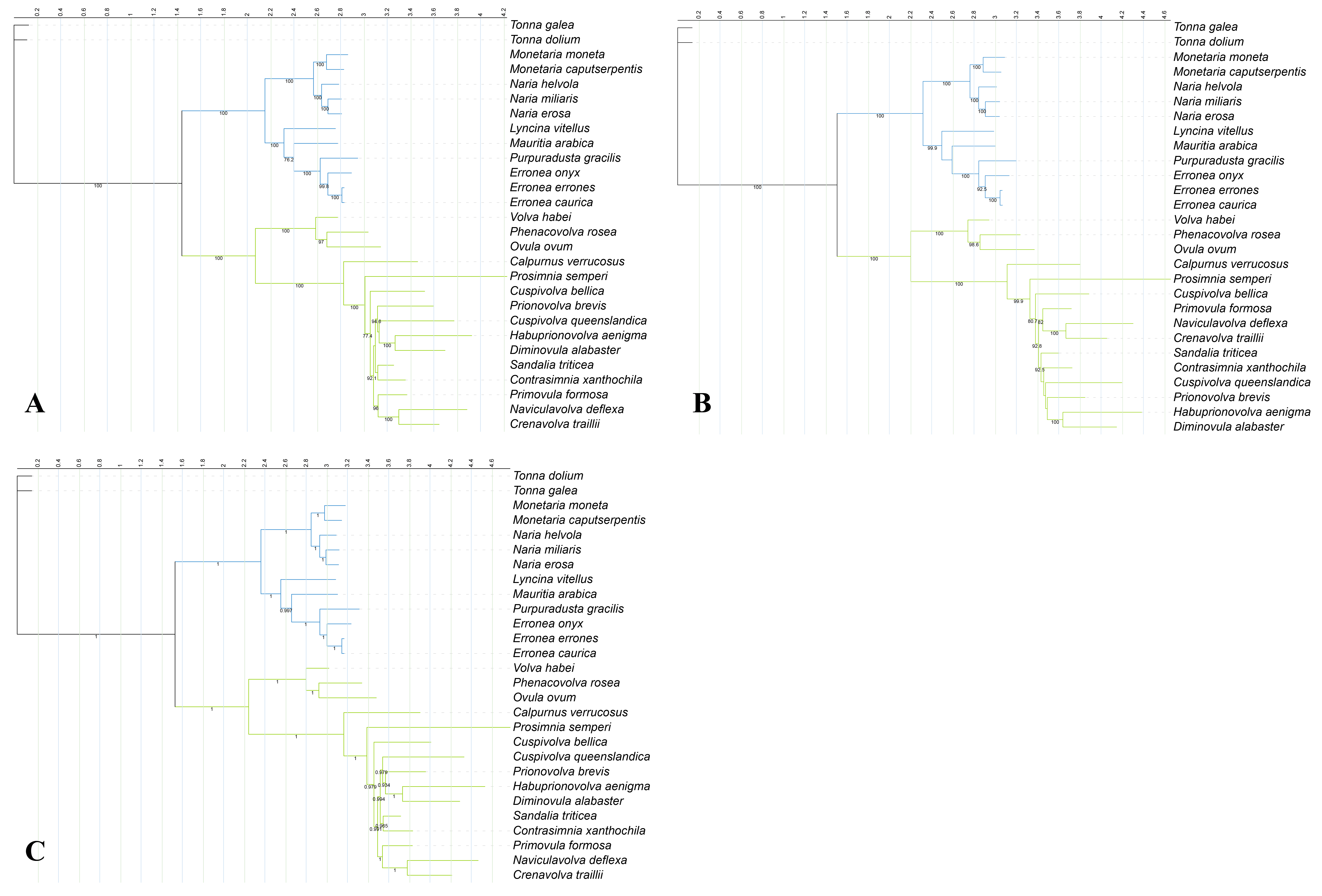

Supplement: Supplementary file 1 — Figure S1. Mitophylogeny for families Ovulidae and Cypraeidae. Numbers near the nodes are posterior probability/bootstraps values (only those > 75% are shown). (A) ML phylogenetic tree (13 PCGs + 2 rRNAs) (B) ML phylogenetic tree (13 PCGs) (C) BI phylogenetic tree (13 PCGs). [file ECE3-15-e71224-s002.png]
